# Mitochondrial Metabolism in Carcinogenesis and Cancer Therapy

**DOI:** 10.3390/cancers13133311

**Published:** 2021-07-01

**Authors:** Hadia Moindjie, Sylvie Rodrigues-Ferreira, Clara Nahmias

**Affiliations:** 1 Inserm, Institut Gustave Roussy, UMR981 Biomarqueurs Prédictifs et Nouvelles Stratégies Thérapeutiques en Oncologie, 94800 Villejuif, France; hadia.moindjie@gustaveroussy.fr (H.M.); sylvie.rodrigues-ferreira@gustaveroussy.fr (S.R.-F.); 2 LabEx LERMIT, Université Paris-Saclay, 92296 Châtenay-Malabry, France; 3 Inovarion SAS, 75005 Paris, France

**Keywords:** mitochondria, carcinogenesis, Warburg effect, oncometabolites, ROS, mitophagy, mtDNA mutations, therapy, mitochondrial oxidative respiration, metabolic reprogramming

## Abstract

**Simple Summary:**

Reprogramming metabolism is a hallmark of cancer. Warburg’s effect, defined as increased aerobic glycolysis at the expense of mitochondrial respiration in cancer cells, opened new avenues of research in the field of cancer. Later findings, however, have revealed that mitochondria remain functional and that they actively contribute to metabolic plasticity of cancer cells. Understanding the mechanisms by which mitochondrial metabolism controls tumor initiation and progression is necessary to better characterize the onset of carcinogenesis. These studies may ultimately lead to the design of novel anti-cancer strategies targeting mitochondrial functions.

**Abstract:**

Carcinogenesis is a multi-step process that refers to transformation of a normal cell into a tumoral neoplastic cell. The mechanisms that promote tumor initiation, promotion and progression are varied, complex and remain to be understood. Studies have highlighted the involvement of oncogenic mutations, genomic instability and epigenetic alterations as well as metabolic reprogramming, in different processes of oncogenesis. However, the underlying mechanisms still have to be clarified. Mitochondria are central organelles at the crossroad of various energetic metabolisms. In addition to their pivotal roles in bioenergetic metabolism, they control redox homeostasis, biosynthesis of macromolecules and apoptotic signals, all of which are linked to carcinogenesis. In the present review, we discuss how mitochondria contribute to the initiation of carcinogenesis through gene mutations and production of oncometabolites, and how they promote tumor progression through the control of metabolic reprogramming and mitochondrial dynamics. Finally, we present mitochondrial metabolism as a promising target for the development of novel therapeutic strategies.

## 1. Introduction

Carcinogenesis is the process by which a normal cell evolves until it becomes a cancerous cell. The etiology of carcinogenesis is complex, multifactorial and can involve cellular, molecular, genetic, epigenetic and environmental alterations [1]. Among the six hallmarks of cancer that were updated by Hanahan and Weinberg in 2011, the deregulation of cellular energetics stands as a major mechanism supporting neoplastic transformation [2]. In cancer, metabolisms of amino acids, lipids, nucleic acids, glutamine and glucose can be altered to promote cell proliferation and survival [3,4]. Rewiring cell metabolism is one way by which cancer cells survive to gene alterations, low nutrients availability, hypoxic environment and increased stiffness of surrounding tissues. Cancer cells thus reprogram their metabolism to gain energetic metabolites that fuel cancer initiation and maintenance [5].

Mitochondria are major players in metabolic reprogramming of cancer cells. These organelles, often qualified as “the powerhouse of the cell”, have been the subject of intense research over the past 50 years because of their pleiotropic functions. Long been considered as the center of cellular energy production, mitochondria are more than the energetic factories of the cell [6]. They constitute an integrative hub controlling ATP generation, amino-acid synthesis, ROS production, redox balance, calcic signaling and apoptotic pathways [7,8]. Mitochondria also represent “stress sensors” that coordinate metabolic adaptation of cells to their microenvironment.

In this review, we will present the major metabolic pathways linked to mitochondrial functions in normal cells. We will also review the impact of mitochondrial gene mutations in the initiation of carcinogenesis and the implication of metabolic reprogramming and mitochondrial dynamics in the maintenance of cancer. Finally, we will discuss the possibility of targeting mitochondrial metabolism as new promising cancer therapeutic strategies.

## 2. Mitochondria, the Powerhouse of the Cell

Mitochondria are inherited maternal organelles of round to oval shape, ranging in size from 0.5 to 3 micrometers, that are localized in the cytoplasm of eukaryotic cells. These organelles are bordered by an outer membrane (OMM) that connects mitochondria with other organelles and constitutes a platform for exchanges—of small ions, metabolites, nucleotides and proteins—between mitochondria and the cytoplasm [9,10,11]. The OMM surrounds the inner membrane (IMM), a highly impermeable structure comprising many membrane invaginations called cristae. Mitochondrial matrix, the inner space delimited by IMM, contains the mitochondrial genetic material. Mitochondrial DNA (mtDNA) consists in a small, 16.6 kb long, double-stranded and circular DNA containing 37 genes that encode 13 proteins implicated in the mitochondrial respiratory chain. mtDNA also generates 22 transfer RNAs and 2 ribosomal RNAs required for mitochondrial protein synthesis machinery [12,13]. There are around 1500 mitochondrial proteins. Among them, only 1% are encoded by mtDNA, the remaining 99% being encoded by nuclear genes, suggesting a close communication between mitochondria and the nucleus.

**Figure 1:**
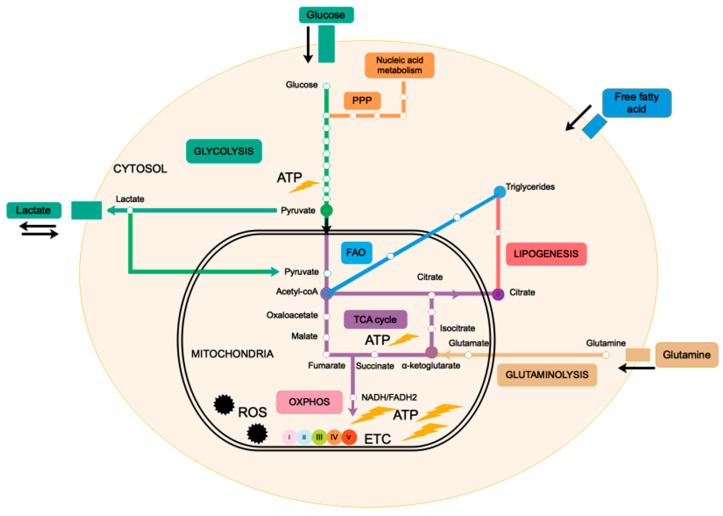
Mitochondrial metabolic pathways. Catabolism of glucose, glutamine and fatty acids all contribute to mitochondrial energetic metabolism. Filled circles represent the intersections between mitochondrial and cytosolic metabolic pathways. Cellular ATP is mainly produced in mitochondria through oxidative respiration that depends on cytosolic glycolysis and mitochondrial TCA cycle. Mitochondrial citrate produced in the TCA cycle contributes to lipid synthesis in the cytosol. TCA cycle and OXPHOS are fueled by pyruvate, glutamate and acetyl-coA produced by glycolysis, glutaminolysis and fatty acid β-oxidation (FAO), respectively. Glycolysis can also contribute to nucleic acid metabolism via the pentose phosphate pathway (PPP). Extracellular lactate can be oxidized in mitochondria and is converted into pyruvate, thereby fueling oxidative respiration to produce ATP.

Mitochondria produce cellular energy in the form of adenosine triphosphate (ATP) through oxidative phosphorylation (OXPHOS), a process that occurs in the electron transport chain (ETC) under aerobic condition. During oxidative respiration, mitochondria consume cellular oxygen and produce reactive oxygen species (ROS). In an anaerobic condition, ATP is mainly produced by glycolysis in the cytosol. Cellular respiration produces ATP molecules from glucose catabolism by three interconnected metabolic pathways: glycolysis, tricarboxylic acid (TCA) cycle and OXPHOS. Briefly, glucose enters in the cell via specific transporters (GLUTs) and is oxidized to pyruvate during glycolysis. Pyruvate is then transported into the mitochondria and enters the TCA cycle in the form of acetyl-coA. In addition to pyruvate, the TCA cycle in the mitochondrial matrix can also be fueled by intermediate metabolites such as glutamate and acetyl-coA produced by glutaminolysis and fatty acid β-oxidation (FAO), respectively (Figure 1). The TCA cycle generates the NADH and FADH2 electron transporters, which supply the ETC localized in the IMM. ETC complexes (I–V) are composed of multiple enzymes that create a proton gradient used by ATP synthase (complex V) to generate ATP [14]. Glycolysis and TCA cycle generate each 2 ATP molecules per glucose molecule, while OXPHOS generates 36 ATP molecules per glucose molecule, making oxidative respiration 18-times more profitable than glycolysis under aerobic conditions. Until recently, it was believed that the final end-product of glycolysis was lactate, which is secreted in the extracellular microenvironment. It was then shown that in some instances, lactate dehydrogenase (LDH)—an enzyme that catalyzes the reversible oxidation/reduction of lactate and pyruvate—allows lactate conversion into pyruvate. Pyruvate then enters in the mitochondrion where it is oxidized to generate ATP through OXPHOS [15,16]. Thus, in these particular cases, glycolysis appears to contribute to OXPHOS. All these metabolic pathways are summarized in Figure 1. The ability of mitochondria to use various sources of carbon to produce energy allows cells to switch between different metabolic pathways in response to variations in the microenvironment. This adaptative property places mitochondria at the center of metabolic flexibility, which is not only essential to cellular homeostasis but also represents a major mechanism of carcinogenesis.

**Figure 2:**
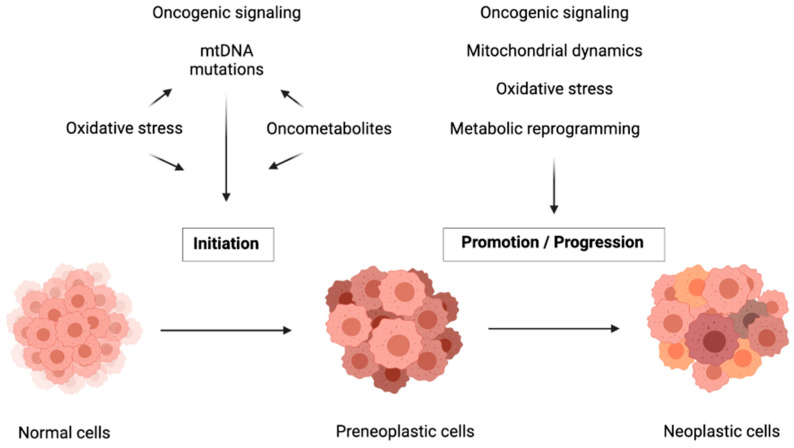
Carcinogenesis is a multistep process to which mitochondria contribute in various ways. Mitochondrial gene mutations are crucial for tumor initiation and mitochondrial-driven regulation of metabolic reprogramming is necessary for tumor promotion and progression.

The pleiotropic implications of mitochondria in cancer have been recently reviewed [17]. Mitochondria contribute to carcinogenesis by regulating cell metabolism and oxidative stress. Dynamics of fusion and fission, mitophagy and dialogue with other organelles are additional ways by which mitochondria participate in the process of cancer. Of note, different mitochondrial dysfunctions are associated with each step of tumor progression (Figure 2). In the initiation of carcinogenesis, mitochondrial ROS promote the transformation of normal cells to preneoplastic cells mainly through oncogenic mtDNA and nuclear DNA mutations that alter cell respiration and promote oncometabolites accumulation and activation of oncogenic pathways. Later stages of tumor progression are rather associated with mitochondrial metabolic reprogramming stimulated by oncogenes, mitochondrial dynamics and oxidative stress.

## 3. Mutations in Genes Involved in Mitochondrial Metabolism Drive Carcinogenesis Initiation

The causal link between alteration of mitochondrial metabolism and initiation of carcinogenesis is illustrated by the occurrence of mutations in mtDNA and nuclear-encoded mitochondrial genes that alter oxidative respiration and promote oxidative stress and epigenetic processes.

### 3.1. Mutations and Decreased Copy Number of mtDNA

Mutations in mtDNA and decreased mtDNA copy number are frequently found in cancer cells and are believed to drive carcinogenesis [18]. Driving effects of mtDNA mutations in carcinogenesis initiation have been clearly established in cancer cell lines depleted for mtDNA (ρ0 cells), where introducing an mtDNA mutation was found to promote cancer growth and ROS production [19]. mtDNA is more often mutated than nuclear DNA, probably due to lack of histone protection, limited capacity of DNA repair and proximity with the respiratory chain which is the major producer of ROS [20]. Indeed, mitochondrial ROS induce oxidative damage to lipids and proteins and mutagenize mtDNA, thereby coupling respiratory chain deficiency and carcinogenesis [21]. Mutations of mitochondrial genes encoding NADH dehydrogenase (component of complex I), cytochrome B (complex III), COX I (complex IV) and ATP synthase (complex V) have been associated with a deficit of respiratory function, together with lactate accumulation and ROS overproduction which further stimulates oncogenic signaling pathways [19,22,23,24]. mtDNA mutations in cancer have been extensively reviewed and listed by Hertweck and Dasgupta in 2017 [25]. Elevated percentage of mtDNA mutations is often correlated with high degree of bioenergetic defects. Given that there are hundreds of copies of mtDNA in each cell, mutations can affect all mtDNA molecules (homoplasmy) or a variable proportion of mtDNA molecules (heteroplasmy) which leads to different disease phenotypes [26]. For instance, homoplasmic mutations of ND5 protein (complex I) inhibit tumor growth whereas heteroplasmic ND5 mutations promote tumorigenesis. Indeed, heteroplasmic mtDNA mutations moderately alter mitochondrial functions and promote ROS-dependent cell proliferation whereas homoplasmic mutations induce severe mitochondrial damages with lethal consequences for cancer cells [23]. In addition to mutations in the mtDNA coding regions, highly frequent somatic mutations occur in a non-coding region of mtDNA called the D-loop, that constitutes a mutational “hotspot” [25]. The D-loop region controls mtDNA replication and transcription. Mutations in this region decrease mtDNA copy number and thus alter expression of mitochondrial genes, with deleterious consequences for mitochondrial integrity and a promoting effect in carcinogenesis [18]. Importantly, mtDNA depletion is also correlated with both hyper- and hypomethylation of nuclear genome in a not-yet elucidated epigenetic mechanism [27]. Mitochondrial-driven regulation of the nuclear genome identified with mtDNA mutations highlights a bidirectional interaction between mitochondria and the nucleus.

### 3.2. Mutations in Nuclear-Encoded Mitochondrial Genes

Mutations in mitochondrial enzymes encoded by nuclear DNA also contribute to carcinogenesis in various ways. They compromise the mitochondrial TCA cycle and oxidative respiration and induce ROS production and HIF-1α-dependent pseudohypoxia. Mutations in isocitrate dehydrogenase (IDH), succinate dehydrogenase (SDH) and fumarate hydratase (FH), three important enzymes of the TCA cycle, induce the accumulation of metabolic intermediates, namely 2-hydroxyglutarate (2-HG), succinate and fumarate, that are called mitochondrial oncometabolites. These three oncometabolites are significantly increased in tumor cells compared to normal cells [28,29]. Accumulation of oncometabolites promotes initiation of carcinogenesis by altering mitochondrial-dependent biosynthesis pathways, redox balance and regulating nuclear genome epigenetic processes.

#### 3.2.1. Bioenergetic Metabolism Alteration

IDH, SDH and FH mutations all induce a decrease in mitochondrial respiration because of a blockade of the TCA cycle and OXPHOS. As a result, these mutations are associated with an upregulation of glycolysis and lactate production [30,31]. Oncometabolites promote glycolysis mainly by inhibiting pyruvate dehydrogenase (PDH), a key enzyme that converts mitochondrial pyruvate to acetyl-coA, the first intermediate that enters the TCA cycle. PDH inhibition interrupts the TCA cycle but also prevents HIF-1 degradation, resulting in HIF-1 accumulation that induces expression of genes involved in glycolysis [32,33]. In response to mitochondrial bioenergetic decrease, compensatory pathways can be activated, such as glutaminolysis in IDH-1 mutated glioma, to maintain metabolic homeostasis [34].

#### 3.2.2. Oxidative Stress Promotion

Oncometabolites were also shown to disrupt redox homeostasis, which depends on the balance between production of ROS (superoxide anions, H_2_O_2_, hydroxyl radicals) and antioxidant pathways. In particular, SDH being both a TCA cycle enzyme and a component of ETC complex II, its mutations were shown to block electron transport across ETC complexes, leading to overproduction of superoxide anions [35]. Oncometabolites thus promote carcinogenesis in two ways. They either alter ROS scavenging processes by inhibiting Nrf2 transcription factor, depleting intracellular NADPH and/or compromising gluthatione (GSH) disulfide reduction—all antioxidant systems that protect cells from oxidative stress [36], or they promote ROS generation through alteration of ETC complex II activity [37,38,39]. Interestingly, ROS inhibits PDH, suggesting that oncometabolites may induce a pseudo-hypoxic environment through ROS production. Thus, mutations in mitochondrial enzymes induce a tumorigenic hypoxia-like state mediated by HIF-1 stabilization and accumulation of ROS that promotes tumor growth [40].

#### 3.2.3. Epigenetic Regulation

Another effect of oncometabolites is the induction of carcinogenesis by epigenetic regulation of oncogenes and DNA repair enzymes. Accumulation of 2-HG, succinate or fumarate inhibits α-ketoglutarate-dependent dioxygenases involved in histone and DNA demethylations. They induce histone hypermethylation, resulting in homologous recombination (HR) DNA repair defects through KDM4A and KDM4B inhibition. These two lysine demethylases are necessary for efficient DNA repair and their inhibition leads to genomic instability [41]. Inhibition of demethylases and dioxygenases (TET family) by oncometabolites also mediates epigenetic control of genes implicated in glycolysis [42,43,44]. The implication of mitochondrial functions in the initiation of carcinogenesis are summarized in Figure 2.

## 4. Mitochondrial Metabolic Reprogramming by Oncogenes

In addition to their role in the initiation of carcinogenesis, mitochondria are also described as major players in later stages of cancer progression mainly by reprogramming the bioenergetic cell metabolism. The concept of metabolic reprogramming was proposed a century ago by Otto Warburg, who described the ‘Warburg effect’ in which cancer cells promote aerobic glycolysis and excessive lactate formation rather than OXPHOS to produce ATP even in the presence of oxygen [45,46]. These observations were the first connection between cell metabolism and tumor progression. Long ignored, Warburg’s work became the subject of multiple studies in the 90’s, and it was then generally accepted that metabolic reprogramming and the switch from OXPHOS to glycolysis constitute new hallmarks of cancer [2]. The ability of cancer cells to increase glucose consumption was then largely exploited in the early 2000s with the development of 18F-deoxyglucose positron emission tomography (18F-FDG-PET) to diagnose cancer [47]. However, the question can be raised as why cancer cells promote glycolysis rather than OXPHOS even in the presence of oxygen, when glycolysis has a much lower bioenergetic efficiency than OXPHOS. A first explanation is that in addition to ATP production, glycolysis generates carbon precursors and NAD^+^, thereby promoting the synthesis of nucleic acids, proteins and lipids, that are necessary for fast-growing cells [48]. A second possibility, based on recent studies, highlights a complementary effect of lactate in favor of oxidative respiration in the context of intra-tumoral heterogeneity. Indeed, tumors contain both hypoxic and oxygenated regions. Hypoxic cancer cells produce ATP mainly by glycolysis with a concomitant lactate release. It has been shown that lactate that was released in the extracellular environment by hypoxic cells can be used as a complementary fuel for oxidative respiration in cancer cells that are present in well-oxygenated regions of the tumor. These findings underline the occurrence of mutual metabolic exchanges between cancer cells in hypoxic and better-oxygenated regions in the same tumor to provide their respective access to energetic metabolites [15,49]. A third hypothesis comes from the fact that the speed of ATP synthesis by glycolysis is much greater than by OXPHOS. Thus, in the presence of sufficient nutriments, cancer cells that enhance glucose uptake via increased expression of glucose transporters can produce ATP more quickly. In this way, glycolysis may represent a metabolic advantage to cell growth compared with OXPHOS [50].

Of note, it has recently been recognized that, contrary to what Warburg believed, the promotion of aerobic glycolysis may not be necessarily associated with damaged mitochondrial respiration [51]. Indeed, some cancer cells carry out glycolysis and oxidative respiration concurrently [52] and the capacity of cells to switch reciprocally from OXPHOS to glycolysis now appears as a key mechanism of metabolic plasticity [51,53,54].

Metabolic reprogramming is finely regulated by an oncogenic triad comprising HIF-1, MYC and p53 mutants that mainly enhance glycolysis [55]. They increase the expression levels of key proteins of the glycolytic pathway such as glucose transporters (GLUT1, GLUT3), hexokinases that convert glucose into glucose-6-P and lactate dehydrogenase (LDHA) that reversibly converts pyruvate into lactate [56,57,58]. These three oncogenes also interrupt oxidative respiration by inhibiting PDH. Wildtype p53 positively regulates OXPHOS through upregulation of cytochrome c oxidase SCO2, a component of the ETC complex IV, and by inducing the expression of TIGAR, a regulator of glycolysis and apoptosis [59]. Not surprisingly, mutations of the TP53 gene are frequently associated with a Warburg effect. However, TP53 mutations were also shown to promote OXPHOS, which further underlines p53-dependent metabolic plasticity [60].

## 5. Mitochondrial Metabolic Reprogramming in the Progression of Carcinogenesis

Mitochondrial metabolic reprogramming in cancer cells is also regulated by mitochondrial dynamics that depends on cycles of fusion–fission, on the balance between mitochondrial biogenesis and degradation through mitophagy, as well as on the crosstalk between mitochondria and the nucleus.

### 5.1. Mitochondrial Dynamics

Mitochondria are dynamic organelles that form an interconnected tubular network. Network morphology is regulated by alternance between phases of fusion and fission, which model the shape of mitochondria [61]. Mitochondrial fusion results in the joining of two adjacent mitochondria in a single elongated organelle, whereas fission generates two fragmented mitochondria out of one. Mitochondrial fission is essential to maintain adequate numbers of mitochondria in dividing cells and removing damaged mitochondria, while fusion is promoted by energy demand and stress and allows transfer of contents. Fusion between damaged and intact mitochondria allows transfer of RNA, protein components or mtDNA in the form of nucleoid in a complementation mechanism [62,63]. Mitochondrial morphology is regulated by GTPases, including the dynamin-related-protein-1 (Drp1) that regulates mitochondrial fission, and the mitofusin family of proteins (Mfn1, Mfn2) and optic atrophy 1 (Opa1) that stimulate mitochondrial fusion. Interestingly, Mfn2 and Drp1 expressions are regulated by p53, providing a link between mitochondrial fusion/fission and carcinogenesis [64]. The correlation between mitochondrial dynamics and metabolism was first suggested in 1966 with the study of mitochondria isolated from mouse liver. In this pioneer study, Hackenbrock et al. described reversible changes of mitochondria ultrastructure according to metabolic steady states [65]. Since then, many studies have described reduced oxidative respiration in fragmented mitochondria compared to elongated mitochondria [66,67]. It is now clear that mitochondrial architecture and metabolic functions are closely related, as mitochondria adapt their morphology in response to cell microenvironment and nutrient conditions to ensure cell survival [68]. Indeed, in a rich-nutrient environment mitochondria are in a fragmented state, while under starvation conditions mitochondria are in an elongated form [69,70]. Dysregulation of mitochondrial dynamics with an increase in mitochondrial fission has been observed in many cancers [71,72,73]. Excessive mitochondrial fission induced by invalidation of Opa-1, Mfn1 and Mfn2 or by activation of Drp1 generates fragmented mitochondria, which highly activates glycolysis. Interestingly, mitochondrial fragmentation induced by an excess of fission also potentiates ROS production [74,75], due to mitochondrial membrane depolarization [76,77]. In turn, mitochondrial ROS induce post-translational modifications of Drp1, Mfn and Opa-1 with subsequent damage in mitochondrial morphology and functions, forming a feedback loop [78,79]. Together, these findings further illustrate metabolic flexibility in carcinogenesis.

### 5.2. Mitophagy

In addition to mitochondrial dynamics, the regulation of mitochondrial mass—a surrogate marker of the quantity of mitochondria per cell—is pivotal in mitochondrial function and in tumorigenesis [80,81,82]. Mitochondrial mass homeostasis depends on a balance between biogenesis and degradation. Supernumerary or defective mitochondria are eliminated by a selective autophagy process called mitophagy. Mitophagy represents the quality control of mitochondrial function. It can be promoted by two pathways: a PINK1-Parkin-mediated ubiquitin pathway and a hypoxia-mediated mitophagy process dependent on receptors (BNIP3, BNIPL3/NIX and FUNDC1), and independent of PINK1. PINK1-mediated mitophagy is activated upon mitochondrial membrane depolarization, oxidative stress or mtDNA mutations as signals of mitochondrial defects. In PINK1-dependent mitophagy, damaged mitochondria induce the accumulation of PINK1 on the OMM, and the recruitment and activation of Parkin from cytosol. Parkin ubiquitinates OMM proteins and thus induces degradation of damaged mitochondria by autophagy. In the second pathway, BNIP3, NIX and FUNDC1 expression are induced by hypoxia. Damaged mitochondria increase the expression of BNIP3, NIX and FUNDC1, a family of mitophagy receptors which are localized in the OMM [83] and directly recruit LC3 through their LC3-interacting region (LIR) to initiate mitophagy [84,85,86]. Mitophagy plays opposite roles in tumorigenesis according to tumor type, stage of carcinogenesis and the context of tumor microenvironment. This process can support cancer cell survival through elimination of damaged mitochondria or act like a tumor suppressor by eliminating damaged mitochondria that otherwise may promote carcinogenesis. In general, in the initiation of carcinogenesis, mitophagy is inhibited through Parkin mutations while during tumor progression, mitophagy is increased via abnormal regulation of BNIP3. This tumor adaptation may stand as a mechanism to increase cancer survival [87]. Loss of mitophagy induces accumulation of damaged mitochondria and stimulates carcinogenesis, as exemplified by parkin-null mice which develop spontaneous hepatocellular carcinoma [88]. Interestingly, silencing of BNIP3 leads to accumulation of dysfunctional mitochondria and ROS overproduction that stimulates HIF1-target genes involved in glycolysis promotion and carcinogenesis [89]. In contrast, studies conducted in hepatocellular carcinoma revealed that hepatitis B virus induces BNIP3L-dependent mitophagy that upregulates glycolytic metabolism to increase HCC cell growth [90]. This dichotomic effect of BNIP3 as a tumor-suppressor or an oncogene may be explained by alternative splicing of BNIP3 generating different isoforms of the receptor with opposite effects [91]. Parkin can also indirectly control carcinogenesis by inhibiting HIF-1 transcriptional activity and promoting HIF-1 degradation by ubiquitination [92]. It is important to remember here that the dynamic of mitochondria impacts metabolism and mitochondrial functions but that the converse is true.

### 5.3. Mitochondrial Retrograde Response

Recent studies suggest that mitochondrial retrograde response (MRR), that consists in the transfer of information from mitochondria to the nucleus, occurs in late stages of tumor progression. Mito-nuclear communication is a mechanism hijacked by cancer cells to promote tumor survival through changes in metabolism, stemness, migration and response to cancer treatments [9,93,94].

In conditions of mitochondrial dysfunctions induced by mtDNA mutations, decreased copy number, mitochondrial enzyme defects, OXPHOS alteration or ROS overproduction, mitochondria can send molecular signals to the nucleus to modify nuclear gene expression in order to restore mitochondrial functions. The mechanisms involved in the induction of MRR are not totally understood, but it seems that MRR is induced by modification of mitochondrial potential membrane, and elevation of ROS and calcium levels [95]. It was recently shown that MRR may involve physical contact between mitochondrial and nuclear membranes. These contacts are promoted by translocator protein (TSPO) accumulation on OMM that represses mitophagy and deregulates Ca^2+^ signaling and ATP production [9]. MRR is mediated in part by molecules produced by mitochondria such Ca^2+,^ ATP, ROS, acetyl-coA, NAD+/NADH and oncometabolites. Two Ca^2+^-mediated retrograde signaling pathways have been identified: a Ca^2+/^ calcineurine-mediated MRR that increases nuclear translocation of transcription factors such as NF-κB, NFAT, CREB and C/EBPδ and a Ca^2+^-mediated pathway that depends on protein kinases [96]. In response to stress, these two MRR pathways induce mitochondrial-driven regulation of nuclear genes transcription with profound impact on mitochondrial functions, stress response and/or metabolic reprogramming [93] in the context of carcinogenesis.

Mitochondrial fusion, fission, mitophagy and retrograde signaling are under the control of oncogenes, oxidative stress and metabolites that generate a feed-back loop contributing to the key role of mitochondria in cancer cell adaptability to microenvironment and nutrient stress [97]. Hence, mitochondrial dynamics and mito-nuclear communication represent emerging areas of studies to better understand the driving role of mitochondria in cancer cell adaptation.

## 6. Mitochondria as Promising Targets in Cancer Therapies

**Figure 3:**
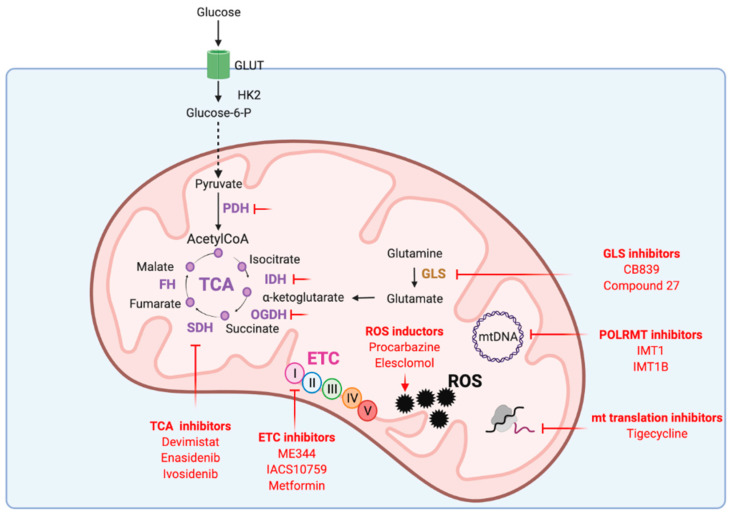
Mitochondrial functions targeted in cancer therapy. ETC: electron transport chain, FH: fumarate hydratase, GLS: glutaminase, GLUT: glucose transporter, HK2: hexokinase 2, IDH: isocitrate dehydrogenase, mtDNA: mitochondrial DNA, OGDH: α-ketoglutarate dehydrogenase, PDH: pyruvate dehydrogenase, POLRMT: mitochondrial RNA polymerase, ROS: reactive oxygen species, SDH: succinate dehydrogenase, TCA: tricarboxylic acid cycle.

Given its key roles in carcinogenesis and tumor maintenance, mitochondria have emerged as interesting therapeutic targets. Addiction of cancer cells to nutrients for their growth have led to consider mitochondrial metabolism as the Achilles’s heel of tumors. Targeting mitochondrial metabolism has thus been widely investigated as potential cancer therapy. Disturbing mitochondrial metabolism and redox balance with pharmacological inhibitors (Figure 3) already gave promising results that are being evaluated in ongoing clinical trials.

### 6.1. Targeting mtDNA Transcription and Translation

As mentioned earlier, mtDNA encodes proteins implicated in mitochondrial respiration. Inhibitors of mtDNA transcription that target the mitochondrial RNA polymerase POLRMT were recently developed [98]. These inhibitors (IMT1 and IMT1B) impair the transcription of components of the OXPHOS system and thus reduce mitochondrial metabolism and ATP level. IMT1 and IMT1B have also been shown to reduce cancer cell growth and viability in vitro and to induce a strong anti-tumor response in ovarian and colon xenograft models [98]. Inhibition of mitochondrial proteins translation was also shown to inhibit cancer cell proliferation and induce cell death [99,100]. For instance, tigecycline, an FDA-approved broad spectrum antibiotic, dose-dependently and specifically inhibits translation by mitochondrial-but not cytosolic-ribosome, thereby leading to oxidative stress and damage, and suppression of mitochondrial respiration [100]. Tigecycline was also shown to increase the efficiency of cisplatin in ovarian cancer cells [100] and of tyrosine kinase inhibitor Imatinib in chronic myeloid leukemia [101]. A phase 1 dose-escalation study of tigecycline administered intravenously to patients with acute myeloid leukemia showed a safety profile [102].

### 6.2. Targeting ETC

Several ETC inhibitors have been shown to disrupt the function of respiratory complexes of the ETC and to induce high levels of ROS that trigger cancer cell death [103]. Most of them target the complex I and are particularly efficient in tumors that rely on OXPHOS for their survival. Promising inhibitors such as metformin, ME344 and IACS10759 are currently in clinical trials. Metformin requires organic cation transporters (OCT) to enter cells and then acts as an anticancer agent through inhibition of mitochondrial NADH dehydrogenase (complex I) [104]. In head-and-neck and breast cancer cells, anti-tumor effects of metformin rely on the expression of OCT [105,106], which may explain the variability of metformin efficiency in clinical trials. OCT expression may thus constitute an appropriate predictive biomarker to identify tumors that are likely to benefit from metformin therapy [107]. Compound ME-344 is a second-generation isoflavone that inhibits mitochondrial NADH biquinone oxidoreductase of complex I [108]. ME-344 also generates ROS, leading to the translocation of Bax to the outer membrane. This translocation induces mitochondrial permeability transition which favors the release of pro-apoptotic molecules [109]. ME-344 was shown to reduce cell growth and viability of AML cell lines and primary AML patient samples, with no effect on normal hematopoietic cells [110]. In a randomized phase 0/1 trial, ME-344 displayed a significant anti-tumor activity on HER2-negative breast tumors [111]. IACS010759 is a small molecule inhibitor that binds a subunit of complex I of the ETC to inhibit electron transfer. IACS010759 efficiency is under evaluation in clinical trials on AML and advanced solid tumors [112,113]. It is to note that in tumors with intact glycolytic system, ETC inhibition may increase glycolysis as an adaptive metabolic response to counteract reduced ATP production and may account for resistance to ETC inhibitors. Thus, combination of IACS10759 with an inhibitor of glycolysis such as 2-Deoxy-glucose (2DG) may represent a useful combination to prevent resistance [114].

### 6.3. Targeting the TCA Cycle

The TCA cycle provides all the biosynthetic precursors necessary for cancer cell growth and maintenance. Indeed, inhibitors of TCA cycle enzymes have shown anti-cancer potential in several cancer types. Among them, CPI-613 (Devimistat) is a lipoate analog that inhibits both α-ketoglutarate dehydrogenase (OGDH) and PDH to prevent the entry of glucose or glutamine-derived carbons into the TCA cycle and alter redox homeostasis [115]. After a safety phase 1 [116], CPI-613 has shown promising results in combination with cytotoxic chemotherapy in a phase 2 study on relapsed or refractory small cell lung carcinoma [117] and in a phase 3 study on metastatic pancreatic adenocarcinoma [118]. Inhibitors of defective enzymes, such as IDH, responsible for 2-HG oncometabolite accumulation, have also been developed. AG221 (Enasidenib) and AG120 (Ivosidenib) efficiently reduce the level of 2-HG and were FDA-approved for IDH-mutated relapsed or refractory acute myeloid leukemia [119]. Inhibition of enzymes that provide pyruvate and glutamate to the TCA cycle are currently under investigation. Targeting glycolysis, either by inhibition of glucose transporters or hexokinase 2, or by using glucose analogs that cannot be metabolized, showed promising results in preclinical studies. However those strategies gave negative results in clinical trials, due to either high toxicity or lack of efficiency [107,119]. Glutamine pathway contributes to ATP production and protein synthesis but also to the control of ROS homeostasis. As for glucose analogs, glutamine analogs have shown severe toxicities, therefore therapeutic strategies have mainly focused on glutaminase (GLS) inhibition. Several inhibitors have been positively evaluated in preclinical studies, showing reduced tumor growth of soft tissue carcinomas, triple negative breast cancers and hematological tumors [120,121,122] and one of them, CB-839 (Telaglenastat), has been evaluated in phase 1/2 trials. A discovery program focusing on optimizing the physicochemical and pharmacokinetic properties of GLS inhibitors has recently been launched [123]. Compound 27 (IPN60090, derived from CB-839) was identified as an orally available and efficient compound in xenograft models and is currently in phase 1 clinical trial [123].

### 6.4. Targeting Redox Homeostasis

Pharmacological increase of ROS level over a toxic threshold has been assessed as cancer therapy. Therapeutic strategies include increased production of ROS or reduced antioxidant response. Indeed, cytotoxic chemotherapies such as cisplatin, 5-Fluorouracil or paclitaxel promote a high level of oxidative stress by increasing ROS. Procarbazine was the first ROS-producing anticancer drug and is approved for the treatment of brain tumors and lymphomas [124]. Elesclomol (STA-4783) is another ROS-generating compound in phase 2 trials for malignant mesothelioma, metastatic melanoma, prostate cancer, advanced kidney cancer and resectable esophageal cancers [124,125]. Elesclomol inhibits super oxide dismutase SOD1 and thus increases ROS by impairing antioxidant defense. Interestingly, delivery of drugs directly into the mitochondria disrupts mitochondrial function and induces mitochondria-dependent apoptosis via rapid generation of ROS [126]. Methods to selectively target mitochondria include the coupling of lipophilic cation such as triphenylphosphonium group (TPP+) to anticancer drugs. Increased mitochondrial transmembrane potential observed in cancer cells favors preferential accumulation of TPP conjugated drugs into cancer cell mitochondria. This new generation of compounds, named ‘mitocans’, efficiently kill multiple types of cancer cells. For instance, mitochondrial targeted vitamin E succinate targeting complex II (MitoVES) and mitochondrial targeted tamoxifen targeting complex I (MitoTAM) efficiently kill colorectal, lung and breast cancer cells and inhibit tumor growth by interfering with complex I-/complex II-dependent respiration without systemic toxicity. Promising results were obtained with MitoTAM tested in phase 1 trial, that is currently extended to phase 2. The promising mitochondrial targets in cancer therapy are presented in Figure 3.

## 7. Conclusions

Almost 100 years ago, Otto Warburg hypothesized that cancer cells promote aerobic glycolysis to produce ATP instead of oxidative respiration which is the main pathway for energy production in normal cells. Warburg then suggested that metabolic reprogramming of cancer cells may be due to irreversible damage of mitochondria. Since then, many studies have shown that contrary to appearances, mitochondria are functional in most cancer cells and actively contribute to carcinogenesis and tumor development.

This review highlights mitochondria as key organelles implicated in all stages of carcinogenesis. Through mtDNA mutations and oncometabolites production, mitochondria favor the initiation of carcinogenesis. Metabolic reprogramming and ROS overproduction, regulated by oncogenes, mitochondrial dynamics and mitochondrial retrograde response, contribute to maintaining the process of carcinogenesis. Although cancer research has focused on the impact of mitochondrial metabolism in cancer cell itself, recent studies suggest that mitochondria may contribute to carcinogenesis in a non-cell autonomous way. Indeed, tumors exhibit metabolic heterogeneity, some cancer cells showing a glycolytic phenotype whereas others are in a more oxidative state depending on nutrients and oxygen availability. Intra-tumoral metabolic crosstalk between cancer cells in hypoxic and oxygenated regions of the tumor may thus contribute to cancer progression.

Production of ROS, lactate and metabolites by mitochondria in cancer cells also impacts the tumor microenvironment. This modified microenvironment in turn induces mtDNA mutations and alters mitochondrial metabolic reprogramming of cancer cells. In line with these observations, a “reverse Warburg effect” has been proposed to partially explain mutual metabolic dependence between the tumor and its microenvironment. This reverse effect is based on the interplay between cancer and stromal cells, which may allow inter-cellular transfer of metabolites [127]. As an example, extracellular lactate produced by cancer associated fibroblasts may be uptaken by cancer cells and be used to fuel mitochondrial oxidative respiration, thereby maintaining carcinogenesis. On the other hand, glycolysis in stromal cells may be induced by tumoral cells. Furthermore, mitochondrial transfer between tumoral and stromal cells has been described as a mechanism that confers an advantage for cancer cell survival [128]. Together, these examples highlight mitochondria as major sources of metabolic exchange between tumor and microenvironment, and new interesting therapeutic targets. Hence, the mechanisms that regulate the mitochondrial metabolic plasticity of tumors represent an active field of research, aiming at developing novel strategies to target the mitochondria of cancer cells.

## Data Availability

Not applicable for a review.
